# Validity and reliability study of anatomy attitude scale in dental students

**DOI:** 10.1186/s12909-025-08418-7

**Published:** 2025-12-09

**Authors:** Kamber Kaşali̇, Didem Özkal Emi̇noğlu, Anvar Dadashov

**Affiliations:** 1 Faculty of Medicine, Department of Biostatistics, Atatürk University, Erzurum, Türkiye; 2 Faculty of Dentistry, Department of Periodontology, Atatürk University, Erzurum, 25000 Türkiye

**Keywords:** Anatomy, Attitude, Dental student, Dentistry

## Abstract

**Background:**

The anatomy education contributes significantly to the cognitive and perceptive learning of students. The purpose of this study was to modify and assess the validity and reliability of the “Anatomy Attitude Scale for Medical School Students”, a tool used to assess medical students’ attitudes about anatomy, for use with dental students.

**Methods:**

The “Anatomy Attitude Scale for Dental Student (AASDS)” scale was tested on 297 students. Subsequently, the completed scale was administered to dental students in order to confirm its validity. Confirmatory factor analysis was used to evaluate construct validity. The reliability of the scale was assessed using test-retest, item correlations, split-half analysis, and Cronbach’s alpha coefficient. The statistical analysis was conducted using package programs. The accepted threshold for statistical significance was *P* < 0.05.

**Results:**

The participants’ average age was 23 ± 2.73 years and 63.4% (*n* = 185) were female. The three-factor structure of the “Anatomy Attitude Scale for Dental Student” as “Value of anatomy”, “Hating anatomy” and “Allocating Time to Anatomy” was confirmed. Cronbach’s alpha coefficient of the scale is 0.588 and McDonald’s ω coefficient is 0.660. It was established that the 14-item scale’s three-factor structure was accurate. Confirmatory factor analysis showed that there was a suitable model for the original version of the scale χ2/df = 2.492, RMSEA = 0.071, SRMR = 0,070, TLI = 0.900 and CFI = 0.922).

**Conclusions:**

The dental students’ version of the “Anatomy Attitude Evaluation Scale for Medical Students” is a valid and reliable instrument for determining dental students’ attitudes towards anatomy.

## Introduction

Anatomy is a fundamental medical study that investigates the organs constituting the human structure and form, the systems created by the integration of these organs, and their interrelationships [1, 2]. The teaching of anatomy contributes significantly to the cognitive and perceptive learning of students. In anatomy education, the integration of sensory experiences and psychomotor activities—such as visual-spatial recognition, model-based exploration, and dissection—enhances students’ cognitive engagement and learning outcomes [3]. Anatomy forms a fundamental component of dental education, providing the structural knowledge necessary for developing the fine motor skills and manual dexterity required in clinical dentistry [4–6]. The anatomy course establishes the foundation for the professional courses that students will undertake in subsequent years. For this reason, students should be very well trained in the subjects given in this course [7, 8]. Anatomy education is carried out in health-related institutions and colleges in the form of theoretical and practical course hours within the possibilities of the institutions. The aim of this course is to enable students studying in areas related to human health to learn the structure of the parts of the body and their relationships with each other. The contents of the courses are transferred to the students theoretically and supported by practice in practical courses. Cadavers, models, plastinated materials, anatomical modelling, radiological images, mobile augmented reality applications are used as practical course materials [4, 9–12]. The anatomy course, which starts to be taught in the first year of education, causes negative prejudices due to its intensive anatomical terminology. Anxiety manifests in students, adversely affecting their interest in the anatomy course and their ability to engage with subsequent coursework. Determining the views and attitudes of dental students about anatomy may be of great importance as a guide in preventing such possible negativities.

Attitude is an individual’s assessment of other persons, things, or ideas [13, 14]. Attitudes have three elements: the cognitive component, which includes ideas and beliefs about the object; the emotional component, which encompasses emotional reactions towards the object; and the behavioral component, which pertains to behaviors directed towards the object [15]. During the literature study, it was discovered that there is no “Anatomy Attitude Scale” that directly indicates the attitudes of dentistry school students toward anatomy and was established specifically for dental students. This condition justifies the creation of a measurement instrument that collects relevant and reliable data to evaluate dentistry students’ views regarding anatomy.

Anatomy education for dental and medical students is an extremely important for students in both fields. Although anatomy lessons are offered in both fields, there are some important differences in the content, scope, and evaluation methods. These differences arise because students in both fields may need different knowledge and skills. While anatomy education for medical students is aimed at evaluating the entire general anatomical formation of the body, anatomy education for dental students focuses more on head, neck, and tooth anatomy [16]. Anatomy education also constitutes the foundation upon which the motor and cognitive skills of dental students are built. Although manual dexterity and procedural skills are critical in dental training, these competencies depend on a solid understanding of the anatomical structures of the head and neck region. Detailed anatomical knowledge is essential for clinical practices such as local anesthesia, extractions, implant placement, endodontic access, and periodontal surgery, where even minor anatomical misinterpretations can cause complications including nerve or vascular injury. Therefore, anatomy serves as both the theoretical and practical basis for clinical dentistry. Understanding dental students’ attitudes toward anatomy can provide valuable feedback for improving educational strategies and ensuring safer, anatomy-based clinical performance [4–6].

The objective of this research is to adapt the “Anatomy Attitude Scale for Medical School Students,” which was established by Mehmet Ali Can in 2022 for students of the faculty of medicine, in a valid and reliable manner to show attitudes toward anatomy for dental students. In the light of the data to be obtained, new educational programmes can be developed to prevent students’ attitudes towards anatomy education, possible concerns and prejudices.

## Materials and methods

### Study design

The study is a two-stage observational validation study. In the first stage, Mehmet Alı Can was contacted via e-mail and the necessary permission was obtained for the adaptation of the scale for dentistry students [17]. Then, the necessary permission for the study was obtained from “Atatürk University Faculty of Dentistry Ethics Committee (No: 49, Date: 25/10/2023, Session number: 10/2023)”. The study followed the guidelines of the “Declaration of Helsinki”. Informed Consent obtained from all the participant.

### Sample size

When type 1 error = 0.05, type 2 error = 0.20 (Power = 0.80) and Cronbach’s alpha is 80%, the minimum sample size required for our study is 180. Considering a 10% loss, this number has been set at 200.

### Data collection tools

The data collecting instrument (survey) comprised sociodemographic questions such as age, gender, reasons for selecting the faculty of dentistry for dental students, and an anatomy attitude scale student questionnaire.

### Anatomy attitude scale for medical school students

The scale is a 14-item self-assessment measure for evaluating medical students’ attitudes about anatomy [17]. The measure uses a 5-point Likert scale: strongly disagree (1), disagree (2), slightly agree (3), agree (4), and strongly agree (5). Scale objects are graded from 1 to 5 points. No items are scored in reverse. The scale includes three dimensions: (1) The importance of anatomy, (2) disliking anatomy, and (3) devoting time to anatomy. The total score received from the scale ranges from 14 to 70. Dimension ratings range from 7 to 35 points for the value of anatomy, 4 to 20 points for hating anatomy, and 3 to 15 points for allocating time to anatomy.

### The procedures performed within the scope of adapting the scale to dentistry students

The “Anatomy Attitude Scale for Medical School Students” established for medical students was reviewed by two dentists, and only minor alterations were made to the questions for dentistry students. The “Anatomy Attitude Scale for Medical School Students” was first translated from English to Turkish, and then back to English. Then, the obtained form was reviewed by a language expert in terms of meaning and grammar. After the expert approval, the “Anatomy Attitude Scale for Dental Students” form was created for dental students and presented in Table 1.

**Table 1:** Dental students version of “Anatomy attitude scale for medical Students”

Items	Factors
Item1	If I were the minister of health, I would abolish the anatomy course from dentistry faculties.*
Item2	Learning anatomy makes me happy.
Item3	I would not call a person who does not know anatomy a dentist.
Item4	If the most unnecessary courses are ranked, “anatomy” comes first.*
Item5	Anatomy information should be reminded at the beginning of each internship.
Item6	Recognising the human body with “anatomy” makes me feel like a dentist.
Item7	If I were in charge, I would remove anatomy information from the “Dentistry Speciality Examination”.*
Item8	If I were a dental education planner, I would recommend anatomy only as an elective course.*
Item9	I would like to do a PhD in anatomy when I graduate.
Item10	Drawing anatomical figures makes me happy.
Item11	I watch anatomy videos in my free time.
Item12	Anatomy practical lessons are interesting.
Item13	I liked anatomy very much because of the lecturers.
Item14	Anatomy is the basis of other dentistry courses.

* Items written have negative meanings and scored reversely

A pilot study involving 25 dentistry students was carried out prior to data collection. The dental students who participated in the pilot study were selected from all years (1st, 2nd, 3rd, 4th, and 5th years). It was determined whether the students who participated in the pilot study understood the questions correctly. Then the actual data collection process started and the results of the students who participated in the pilot study were not included in the actual study.

Three hundred students were invited to the study. The study sample consisted of dental students enrolled in all academic years (1st to 5th year) at the Faculty of Dentistry, Atatürk University. The dental students who answered the questionnaire were asked to answer the same questionnaire again after 3 months.

### Statistical analyses

The scale’s validity and reliability were examined using the statistical packages JAMOVI 2.2.2, AMOS v20.0 (Analysis of a Moment Structure), and SPSS v20.0 (Statistical Package for Social Sciences). Descriptive statistics are provided for the demographic data. The sociodemographic features were displayed as percentages or as mean ± standard deviation (SD). Scores on a scale are displayed as mean ± SD. Data analysis was performed using the Pearson correlation coefficient to examine the relationships among the sub-dimensions of the scale. The normality of each sub- dimension was assessed using the Shapiro–Wilk and Kolmogorov–Smirnov tests, and all sub-dimensions were found to be normally distributed. Therefore, the assumptions for applying the Pearson correlation were satisfied. The Cronbach’s alpha coefficient, McDonald’s omega, split half, correlation between forms, Guttman split-half coefficient, and Hotelling’s T-squared findings are provided for the scale’s internal consistency. Kaiser-Meyer-Olkin (KMO), Bartlett sphericity tests, Scree plots, and correlation matrices were employed to assess the construct validity of the scale. Furthermore, the correlation between the test and test-retest results was provided. Confirmatory factor analysis was employed to analyze the scale’s fit results. Fit indexes included the Tucker-Lewis index (TLI), Chi-square statistic (χ2), Chi-square degree of freedom ratio (CMIN/DF), root mean square error of approximation (RMSEA), standardised root mean square (SRMR), and comparative fit index (CFI). With a path diagram, the confirmatory factor analysis model is displayed. A *p*-value of less than 0.05 was deemed significant.

## Results

The study included 292 of the 300 students accepted from dental schools (years 1–5). The response rate was 97%.

### Characteristics of participants

The participants’ average age was 23 ± 2.73 years (18–28), with 63.4% being female. Table 2 shows the students’ sociodemographic characteristics.

**Table 2:** Sociodemographic characteristics of participants

Sociodemographic characteristics		*N*	%
Gender	Male	107	36,6%
	Female	185	63,4%
Years	Year 1	50	17,1
	Year 2	46	15,8
	Year 3	47	16,1
	Year 4	79	27,1
	Year 5	70	24,0
Why did you choose dentistry?	Because my parents wanted me to.	22	7,5%
	Because this is my ideal career.	76	26,0%
	Because it is a profession that makes money.	44	15,1%
	Because I have enough points.	110	37,7%
	Because it is good status in society	40	13,7%
Do you want to be a dentist specialist in the future?	Yes	225	77,1%
	No	67	22,9%
		Mean ± sd	Med (Min-Max)
Age		23.22 ± 2.73	24 (18–28)

### Findings regarding the validity of the scale

**Fig. 1:**
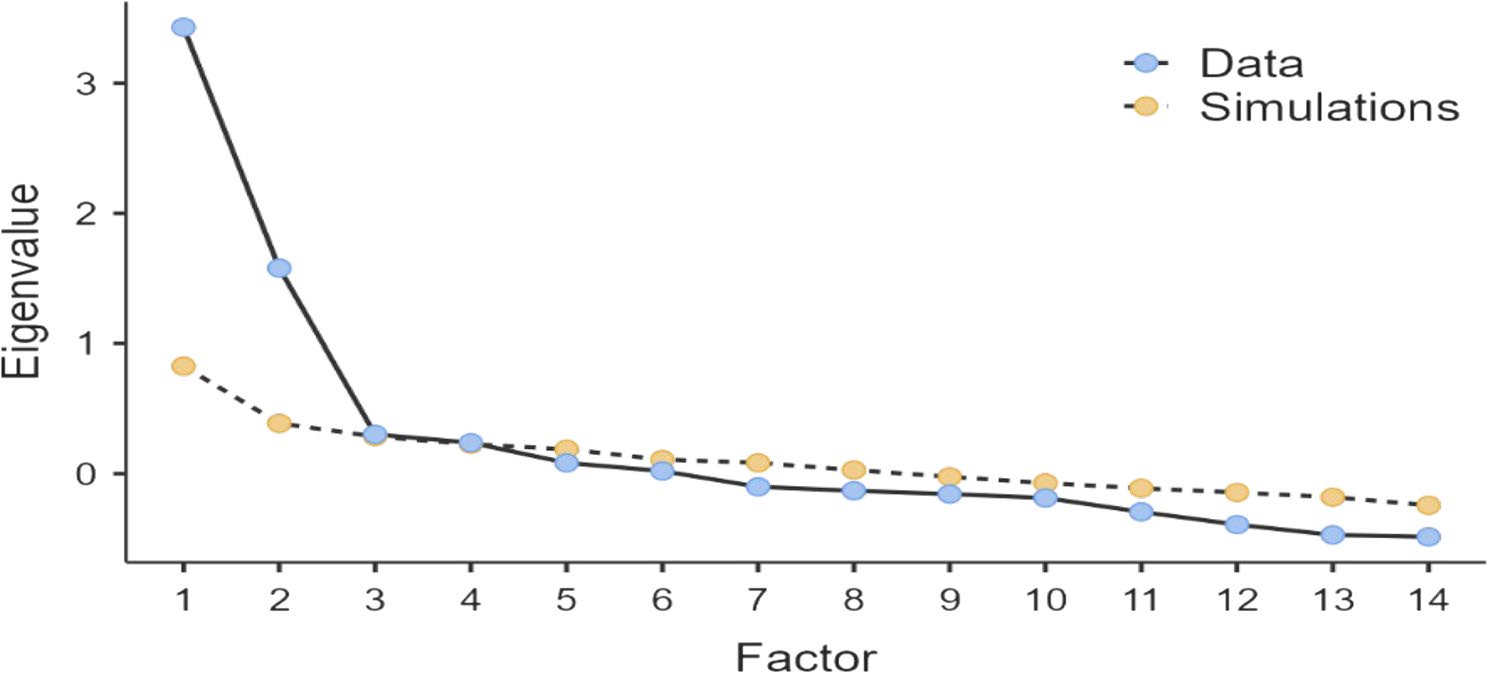
Scree plot for factor analysis of the AASDS

Bartlett’s sphericity test and the Kaiser-Meyer-Olkin measure. The KMO value was 0.807, and Bartlett’s test of sphericity showed statistical significance (approx. = 1203.005, degrees of freedom (DF) = 91, *P* < 0.001). The dispersion point test revealed that the scale included three variables, and factors beyond the third were not explanatory (Fig. 1).

### Confirmatory factor analysis

The CFA model at the first level states, 7 items (items 2,3,5,6,12,13, and 14) are collected in the “Value of anatomy” subdomain, 4 items (items 1,4,7, and 8) in the “Hating anatomy” and 3 items (items 9, 10, and 11) in the “Allocating Time to Anatomy” subdomain (Table 3). Every item that makes up the model contributes in a statistically significant way (*P* < 0.05).

**Table 3:** Sub-dimensions of the “Anatomy attitude scale for dental student (AASDS)”

Items	Factors	Mean ± Sd
	Factor 1: Value of anatomy	21,83 ± 5,38
Item2	Learning anatomy makes me happy.	2,91 ± 1,23
Item3	I would not call a person who does not know anatomy a dentist.	3,40 ± 1,22
Item5	Anatomy information should be reminded at the beginning of each internship.	3,28 ± 1,18
Item6	Recognising the human body with “anatomy” makes me feel like a dentist.	3,73 ± 1,08
Item12	Anatomy practical lessons are interesting.	2,98 ± 1,24
Item13	I liked anatomy very much because of the lecturers.	2,38 ± 1,31
Item14	Anatomy is the basis of other dentistry courses.	3,16 ± 1,26
	Factor 2: Hating anatomy	8,53 ± 3,68
Item1	If I were the minister of health, I would abolish the anatomy course from dentistry faculties.*	1,96 ± 1,23
Item4	If the most unnecessary courses are ranked, “anatomy” comes first.*	2,05 ± 1,28
Item7	If I were in charge, I would remove anatomy information from the “Dentistry Speciality Examination”.*	2,23 ± 1,25
Item8	If I were a dental education planner, I would recommend anatomy only as an elective course.*	2,28 ± 1,22
	Factor 3: Allocating Time to Anatomy	6,44 ± 2,94
Item9	I would like to do a PhD in anatomy when I graduate.	2,10 ± 1,14
Item10	Drawing anatomical figures makes me happy.	2,27 ± 1,26
Item11	I watch anatomy videos in my free time.	2,05 ± 1,15

### Model fit of the scale

**Fig. 2:**
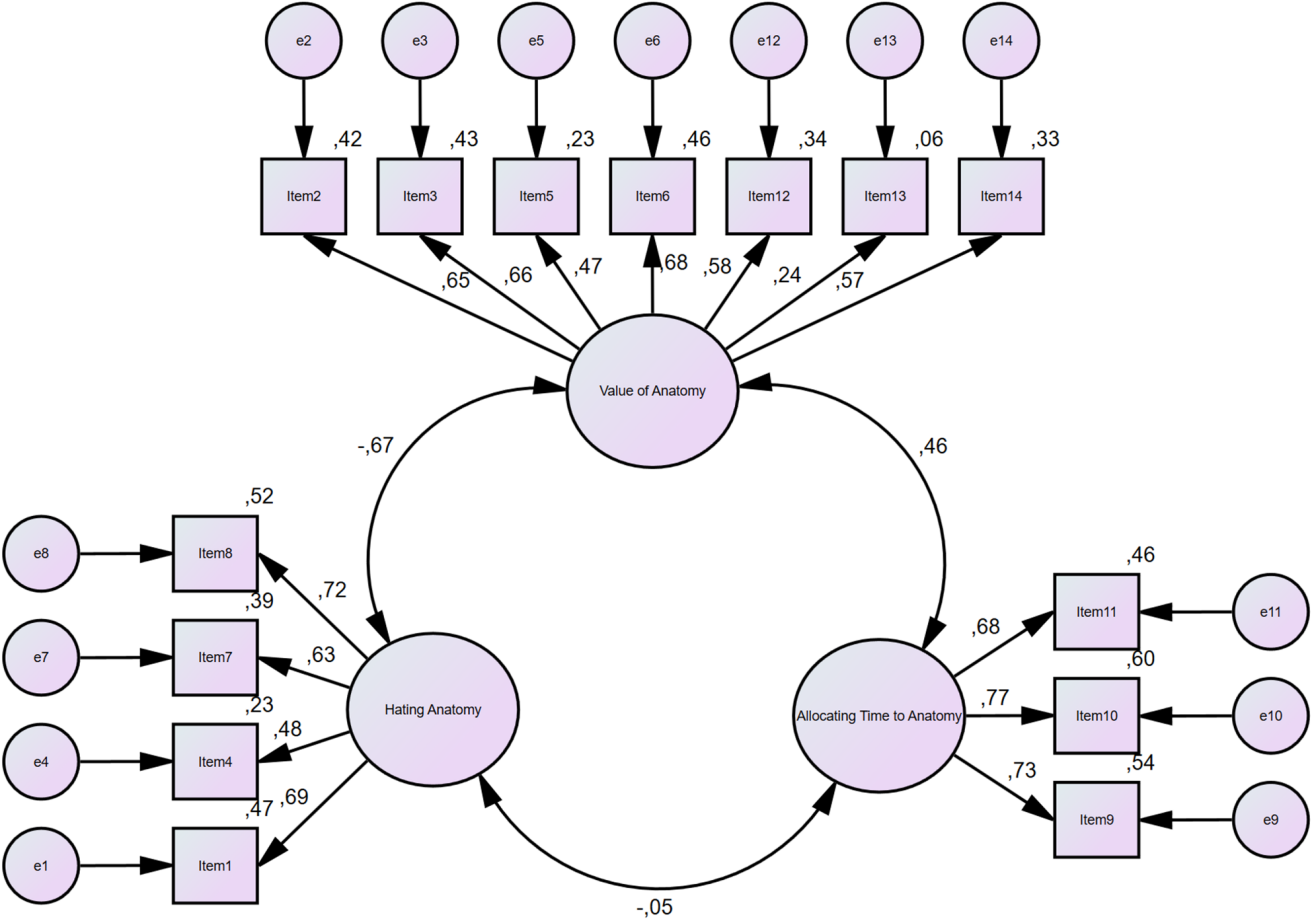
Path Diagram

The first-level CFA model’s goodness-of-fit analysis supported the study’s original design (χ2/df = 2.492, RMSEA = 0.071, SRMR = 0,070, TLI = 0.900, CFI = 0.922). In our investigation, RMSEA, SRMR, TLI, and CFI all demonstrated an adequate fit. Table 4 shows the reference values for the goodness-of-fit analysis findings, as well as the most often used goodness-of-fit indices in the literature (Fig. 2).

**Table 4:** Anatomy attitude scale compliance indexes for dental students

Indexes Reference Value	Good fit	Acceptable fit	Measurement	Result
CMIN/DF	0 < χ 2/Df ≤ 3	3 < χ 2/Df ≤ 5	2,492	Good fit
RMSEA	0 ≤ RMSEA ≤ 0.05	0.05 < RMSEA ≤ 0.08	0.071 (0.057–0.084)	Acceptable fit
SRMR	0 ≤ SRMR ≤ 0.05	0.05 < SRMR ≤ 0.10	0.070	Acceptable fit
TLI	0.95 < TLI ≤ 1	0.90 < TLI ≤ 0.94	0.900	Acceptable fit
CFI	0.95 < CFI ≤ 1	0.90 < CFI ≤ 0.94	0.922	Acceptable fit

*CMIN/DF* Chi-square degree of freedom ratio, *RMSEA* Root mean square error of approximation, *SRMR* Standardised root mean square, *TLI* Tucker-Lewis index, *CFI *Comparative fit index

### Findings regarding the reliability of the scale

**Fig. 3:**
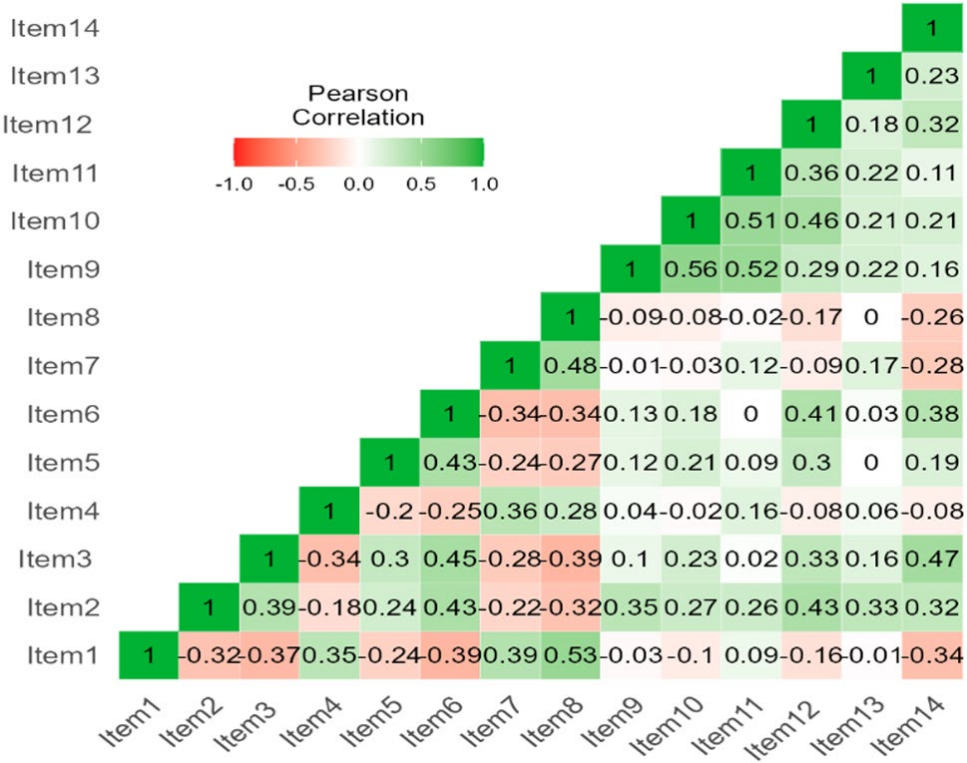
Correlation matrix

Cronbach’s alpha reliability coefficient was 0.588 for the entire scale and 0.745, 0.721, and 0.772 for the values of anatomy, hating anatomy, and allocating time to anatomy, respectively. Cronbach’s alpha values for the retest were 0.538 for the total scale and 0.728, 0.815, and 0.666 for the value of anatomy, hating anatomy, and allocating time to anatomy, respectively. The correlations between test and retest data were statistically significant (Table 5). In the factor, all items are strongly associated (Fig. 3). Cronbach’s alpha scores were 0.746 and 0.622 in the split-half study used to assess the scale’s reliability. Table 6 summarizes the split-half analysis findings. The scale is additive, according to the results of the analysis of variance, which was done to ascertain whether or not the scale items are additive (Nonadditivity: F = 1.875 *P* = 0.171 > 0.05). The measurement variation showed a significant difference (between measures, F = 74.430, *P* < 0.001). Using the Hotelling T2 test, it was determined whether the question means were equal. The results showed a significant difference in the averages (Hotelling’s T-squared = 454.763, F = 33.524, *P* < 0.001).

**Table 5:** The “Anatomy attitude scale for dental student (AASDS)” test and retest reliability

				MEAN	SD	Cronbach’s α	McDonald’s ω	*N* of Items
Test	Value of anatomy			3,120	0,769	0,745	0,758	7
	Hating anatomy			2,130	0,922	0,721	0,727	4
	Allocating Time to Anatomy			2,150	0,983	0,772	0,773	3
	Total			2,630	0,485	0,588	0,660	14
Re-Test	Value of anatomy			2,970	0,838	0,728	0,736	7
	Hating anatomy			2,020	0,996	0,815	0,818	4
	Allocating Time to Anatomy			1,890	0,964	0,666	0,693	3
	Total			2,520	0,475	0,538	0,653	14

				Test				
				Value of anatomy		Hating anatomy	Allocating Time to Anatomy	Total
Re-Test		Value of anatomy	r	0,498		−0,304	0,230	0,331
			p	**< 0**,**001**		< 0,001	< 0,001	< 0,001
			N	268		268	269	267
		Hating anatomy	r	−0,237		0,452	0,008	0,071
			p	< 0,001		**< 0**,**001**	0,891	0,245
			N	268		268	269	267
		Allocating Time to Anatomy	r	0,321		−0,062	0,444	0,421
			p	< 0,001		0,313	**< 0**,**001**	< 0,001
			N	268		268	269	267
		Total	r	0,429		−0,018	0,396	0,511
			p	< 0,001		0,771	< 0,001	**< 0**,**001**
			N	268		268	269	267

*r*: Pearson Correlation, *p *statistical significance level

## Discussion

Although competence in dentistry arises from the integration of cognitive, psychomotor, and affective skills, anatomical knowledge forms the structural foundation upon which manual dexterity and clinical precision are built. Therefore, the Anatomy Attitude Scale for Dental Students (AASDS) evaluates attitudes toward this cognitive component, which indirectly supports psychomotor learning and overall professional development [4–6].

Anatomicy has a profound and antiquated connection to the history of dentistry and medicine. Both fields need understanding of the human body’s structure and functioning. However, there are also significant differences in anatomy teaching and emphasis between the two areas. Within the medical industry, anatomy is commonly seen as a comprehensive discipline that spans the entirety of the human body and is closely connected to clinical practice. Dental medicine was characterized as an integrated, yet distinct and autonomous, medical specialty, in part due to the historical contribution of anatomy to its development. The characterization of the maxillary sinus, salivary glands, tongue and lip muscles, and the microscopic anatomy of dental hard tissues form the foundation of anatomy education in the field of dentistry [5, 6].

The study of anatomy is approached differently by students of dentistry and medicine. The two fields’ approaches to teaching anatomy reflect these distinctions. Anatomy instruction and the application of this knowledge in future professions are advantageous for students in both disciplines [6].

The Anatomy Attitude Scale for Dental Students (AASDS) provides valuable insight into students’ perceptions and attitudes toward anatomy. Such understanding enables educators to recognize areas where students may feel less confident, disengaged, or anxious about anatomy learning. By identifying these areas, curriculum planners and instructors can design targeted educational interventions to enhance motivation and participation in anatomy-related activities. Moreover, understanding students’ attitudes through the AASDS not only benefits anatomy teaching itself but also indirectly contributes to the development of the manual dexterity and psychomotor competencies that are essential for professional dental practice. Overall, this emphasizes the importance of attitude assessment as a pedagogical tool that can guide curriculum development, improve teaching strategies, and ultimately support both cognitive and practical skill acquisition in dental education [4–6].

### Study strengths and limitations

The study’s strength is that it gives researchers with a technique that has been demonstrated to be valid and reliable, as well as results that are comparable internationally. Furthermore, the AASDS is a brief scale that may be used to assess dental students’ attitudes about anatomy. In addition, the findings of this study may contribute to more effective use of time and resources allocated to anatomy in dental education. It can also be used to develop interventions to improve attitudes towards anatomy. However, the following limitations should also be recognised. The AASDS was designed to measure only attitudes towards anatomy. Different scales may need to be used to measure dental students’ knowledge of anatomy or their satisfaction with anatomy courses. During the study, no external factors that may affect students’ attitudes towards anatomy were considered.

## Conclusions

The Anatomy Attitude Scale for dental students has been proven to be a viable and effective tool for measuring their attitudes toward anatomy. The 14-item, three-factor scale’s original structure was maintained throughout the validation process. Thus, the scale may be used as a tool to assess dental students’ ideas on anatomy. Note that the Anatomy Attitude Scale’s validity and reliability for dentistry students can change based on the demographic being examined and the context in which the scale is applied.

## Data Availability

Kaşali, K. (2024). “Validity and Reliability Study of Anatomy Attitude Scale in Dental Students” [Data set]. Zenodo. 10.5281/zenodo.14244844.
